# Liquid Atmospheric Pressure MALDI (LAP‐MALDI) Mass Spectrometry: A Versatile Tool for the Characterization of Synthetic Polymers

**DOI:** 10.1002/rcm.70102

**Published:** 2026-05-14

**Authors:** Agata E. Kowalczyk, Jeffery Brown, Michael Morris, Rainer Cramer

**Affiliations:** ^1^ Department of Chemistry University of Reading Reading UK; ^2^ Waters Corporation Wilmslow UK

## Abstract

**Rationale:**

Liquid atmospheric pressure matrix‐assisted laser desorption/ionization (LAP‐MALDI) mass spectrometry (MS) has previously been applied to the analysis of biopolymers such as peptides, proteins, and DNA, producing ESI‐like multiply charged analyte ions. In this study, LAP‐MALDI MS has been investigated for the analysis of synthetic polymers for the first time.

**Methods:**

A LAP‐MALDI source was interfaced with a Q‐TOF mass spectrometer. The study investigated synthetic polymer standards, including polyethylene glycol (PEG), polypropylene glycol (PPG), polyacrylic acid (PAA), and polystyrene (PS) of different sizes in positive and negative ionization modes with and without ion mobility spectrometry (IMS). The acquired data were compared to ESI MS data using the same polymer standards and instrument but with a standard ESI source.

**Results:**

LAP‐MALDI MS enables the detection of singly and predominantly multiply charged ions with high ion signal stability (< 10% RSD), though with lower charge states than ESI MS. Its flexible sample preparation and ion source setup allow for the controlled manipulation of charge states. Common salts facilitate ionization by metal cation adduct formation and improve detection of PEG, PPG, and PS. PAA was efficiently detected in negative ion mode by deprotonation, and PS required a silver salt for ionization. IMS provides an additional dimension in separation and manipulating charge state distributions, and its data obtained for PEG corroborate previously published ESI IMS results.

**Conclusions:**

LAP‐MALDI MS provides a rapid, less labor‐intensive, and reagent‐efficient way for the analysis of synthetic polymers and produces stable ion signals in both positive and negative ion modes. The source enables the detection of singly and multiply charged ions, with the flexibility to manipulate charge states over a wide range. LAP‐MALDI MS tolerates diverse solvent compositions and offers a versatile platform for high‐throughput polymer structural analysis, including MS/MS analysis that can replace or complement standard polymer analysis tools.

## 1 Introduction

Synthetic polymers are of great interest and use in the manufacturing and consumer industries, with a global market size of almost US$ 800 billion in 2024 [1]. Their tuneable properties make synthetic polymers extremely flexible in their applications across many fields, from packaging and electronics to medicine and aerospace. Easy and low‐cost production, together with their wide range of stability and moldability into different shapes and forms, has made them the material of choice for many products.

For the characterization of synthetic polymers, analytical techniques such as gel permeation chromatography (GPC) [2], nuclear magnetic resonance (NMR) [3], Fourier transform infrared spectroscopy (FTIR) [4, 5], differential scanning calorimetry (DSC) [6], and thermogravimetric analysis (TGA) [7], as well as mass spectrometry (MS) [8] have been commonly used.

GPC is one of the most applied methods and has been historically used as a stand‐alone technique to provide information on molecular weight distribution and the overall size of polymers based on comparing retention times and hydrodynamic radii to values obtained from a reference standard [8]. While GPC is inexpensive and easy to run, it requires excellent quality reference materials and application‐tailored resins and solvent systems. The relationship between retention time and molecular weight is logarithmic, overall making it a good method for analysis of larger polymers (more than several kDa) with a wide degree of polydispersity. Additionally, GPC operates on the assumption that the used reference material is pure and well‐characterized. However, polymers can have different conformations in solution, for example, compact globules or linear coils [9], which will affect their retention times in GPC analysis. If the reference and investigated polymers do not have the same conformation on the GPC column, this can lead to large errors. However, commercially available polymer standards are usually quality controlled with GPC before their release on the market.

In general, GPC analysis of polymers does not offer as much specificity or structural information as many of the MS or NMR methods and often fails to accurately detect lower molecular weight compounds [10]. MS can be employed as a stand‐alone technique for the identification and analysis of polymer properties such as polydispersity index, average mass, monomer, and end group structures [8]. However, some polymers can be difficult to ionize or to keep intact during the MS analysis, which is why different ionization methods can be useful in the MS toolkit.

Most commonly, “soft” ionization techniques such as matrix‐assisted laser desorption/ionization (MALDI), electrospray ionization (ESI), and atmospheric pressure chemical ionization (APCI) are employed for the MS analysis of synthetic polymers [8]. In particular, MALDI has been shown to be well‐suited for the analysis of synthetic polymers [11]. MALDI generates spectra containing predominantly singly charged ions, which are easy to interpret and do not require charge state deconvolution. It provides a quick solution for routine analysis of standard simple polymers. However, conventional (solid) MALDI can suffer from signal instability, “sweet spot” effects, and ineffective crystal formation. Also, singly charged ions are often inferior for MS/MS methods, and large molecules can only be detected by instruments with a high *m/z* range, such as axial TOF instruments [12]. A source producing multiply charged analyte ions, such as an ESI source, can circumvent these restrictions. However, the mass spectra would naturally become more complex—with up to several different charge state distributions that will require rationalization through charge state deconvolution. ESI is also restricted to volatile solvent compositions, which are not always suitable for dissolution of less polar polymers [13]. Additionally, in instruments equipped with orthogonal ion extraction, such as those typically employed with ESI, ions are not sampled uniformly, thus creating an *m/z*‐dependent detection bias [14, 15], which will skew the resulting molecular weight distribution.

Liquid atmospheric pressure (LAP) MALDI, a hybrid soft ionization technique of MALDI and ESI, is capable of analyzing a diverse range of biomolecular classes such as peptides, proteins, and lipids as shown in clinical microbiology and veterinary diagnostics studies [16, 17, 18, 19, 20, 21]. It combines various advantages of laser‐based and atmospheric pressure ionization, providing high analytical speed and sample throughput [22, 23], as well as multiply charged ions [24], high ion signal stability while requiring only small sample volumes (< 1–2 μL) [22, 24], and allowing the interfacing with varied types of high‐performance hybrid instruments such as Q‐TOFs [16, 17, 18, 19, 20, 21, 22, 23] and Orbitraps [25].

In this study, LAP‐MALDI MS is introduced for the analysis of synthetic polymers for the first time, expanding the MS polymer characterization toolkit and demonstrating its unique features.

## 2 Materials and Methods

### 2.1 Materials

Polyethylene glycol (PEG) 3000 (*M*_*n*_ ≈ 3000), polypropylene glycol (PPG) 2700 (*M*_*n*_ ≈ 2700), polyacrylic acid (PAA), and polystyrene (PS) 3000 (*M*_*n*_ ≈ 3000) were sourced from Merck (Gillingham, UK), while PPG 4000 (*M*_*n*_ ≈ 4000) was obtained from Thermo Scientific (Loughborough, UK). *α*‐Cyano‐4‐hydroxycinnamic acid (CHCA) was purchased from Bruker Daltonics (Coventry, UK). 2‐(4‐Hydroxyphenylazo)benzoic acid (HABA), 2,5‐dihydroxybenzoic acid (DHB), dithranol, 3‐nitrobenzyl alcohol (NBA), propylene glycol, silver trifluoroacetate (99.99+%), lithium chloride (anhydrous, ReagentPlus 99%), sodium chloride, sodium acetate (NaAc), potassium chloride, and cesium iodide were bought from Merck. HPLC‐MS grade water (Optima), tetrahydrofuran (THF), acetonitrile, and dimethylsiloxane (DMSO) were purchased from Fisher Scientific in HPLC grade.

### 2.2 Sample Preparation

For LAP‐MALDI, the following matrix chromophore compounds were dissolved in water/acetonitrile (1:1; v:v) at the following concentrations: HABA at ~5 mg/mL (saturated solution), CHCA at 10 mg/mL, and DHB at 20 mg/mL. A solution of the matrix compound dithranol was made at 20 mg/mL in THF, followed by the addition of DMSO at a ratio of 2:1 (v:v; chromophore solution: DMSO). Apart from the dithranol matrix solution with DMSO, propylene glycol was added to all matrix chromophore solutions at a ratio of 5:3 (v:v; chromophore solution: propylene glycol). Polymers and metal salts were dissolved in water/acetonitrile (1:1; v:v) unless they were analyzed using dithranol as matrix compounds, in which case they were dissolved in 100% THF. Polymer solutions were prepared with concentrations of 2–20 mg/mL, and metal salt solutions in a range of 10–1000 mM. Unless stated otherwise, the three (matrix, polymer, and salt) solutions were mixed directly by pipetting 0.5 μL of each on a stainless‐steel, 96‐well target plate (Waters, Wilmslow, UK) and by repeated up and down aspiration.

For ESI, the investigated PEG and PPG polymers were dissolved at 1 mg/mL in water/acetonitrile (1:1; v:v) containing 100 mM of sodium acetate or sodium chloride, while PAA was prepared at 1 mg/mL in water/acetonitrile (1:1; v:v).

### 2.3 MS Analysis

LAP‐MALDI MS analyses were carried out using a custom‐built ion source as described in a previous publication [26], coupled to a Waters SYNAPT G2‐S*i* Q‐TOF mass spectrometer equipped with an 8k quadrupole. A 343‐nm, nanosecond‐pulsed laser (FlareNX 343‐0.2‐2; Coherent, Santa Clara, CA, USA) provided an average beam energy of approximately 5–10 μJ per pulse onto the LAP‐MALDI sample with an incidence angle of ~30° to the sample plate normal, at a rate of 50 Hz and focused by a lens with a 150‐mm focal length. Instrumental parameters were adjusted for each analyte, with an ion extraction potential of 3–4 kV between the sample plate and the heated ion transfer tube, a nitrogen counter‐gas flow of 180–200 L/h, and a cone voltage of 10–150 V. The instrument was calibrated up to *m/z* 5000 using a multipoint calibration with a solution of 10‐mM cesium iodide prepared in the matrix solvent system with propylene glycol as described in Section 2.2 at a nitrogen counter‐gas flow of 0 L/h. The RMS calibration error for the detected cesium iodide cluster ions was < 2 ppm.

For ESI MS, the Waters Lockspray II source was utilized in positive ion mode for PEG and PPG analysis, and in negative ion mode for PAA analysis, using a gas flow of 300 L/h and cone voltages in the range of 60–100 V. The instrument was calibrated for ESI MS analysis using the PEG 3000 standard at 1 mg/mL in 100‐mM sodium acetate.

The data acquisition rate was 1 scan per second, and combining data of 20 scans (20 s) was typically used for data comparison, apart from the PAA analyses where approximately 10 scans (10 s) of data were combined per cone voltage setting. All data were acquired in “TOF” mode unless stated otherwise. The “Mobility TOF” mode was used for some ion mobility analyses of PEG 3000 with a nitrogen gas flow of 220 L/h. Unless stated otherwise, for “Mobility TOF” mode acquisitions, the “Triwave” settings for the wave velocity and height of the IMS, transfer and trap cells were 641 m/s and 29.6 V, 46 m/s and 1.6 V, and 329 m/s and 9.6 V, respectively.

### 2.4 Data Processing

Raw data were collected using MassLynx 4.1 (Waters) and centroided using its automatic peak picking algorithm with default parameters. Peak lists were exported into and normalized by OriginPro 2024b (Northampton, Massachusetts, USA) for spectral plotting. Theoretical isotopic distributions for adducted polymer peaks were calculated using enviPat [27], producing 15‐k FWHM resolution “profiles.” Experimentally obtained polymer peaks were mass‐matched and confirmed with theoretical polymer peaks using MSPolyCalc [28], using a mass tolerance of 30 ppm. Charge deconvolution was carried out with UniDec [29]. In UniDec, the “Deconvolve MS and IM‐MS” mode was chosen without background correction, assuming charge states 1–8+ and sampling data every 0.01 Da with each polymer's accurate monomer mass difference being considered for peak detection. Number‐average molecular weight (*M*_*N*_) and weight‐average molecular weight (*M*_*W*_) values were determined from the obtained charge‐state deconvoluted UniDec data. Ion mobility data were analyzed with DriftScope 2.7 (Waters).

## 3 Results and Discussion

Synthetic polymer analysis by mass spectrometry is typically facilitated by cationization using metal cations from salts such as NaCl, KCl, and AgTFA [11]. Therefore, initial experiments were conducted by adding NaCl solutions at concentrations of 10–1000 mM to the typical LAP‐MALDI sample preparation, mixing the three (matrix, polymer, salt) solutions at a ratio of 1:1:1 (v:v:v) directly on a stainless‐steel sample plate.

**FIGURE 1:**
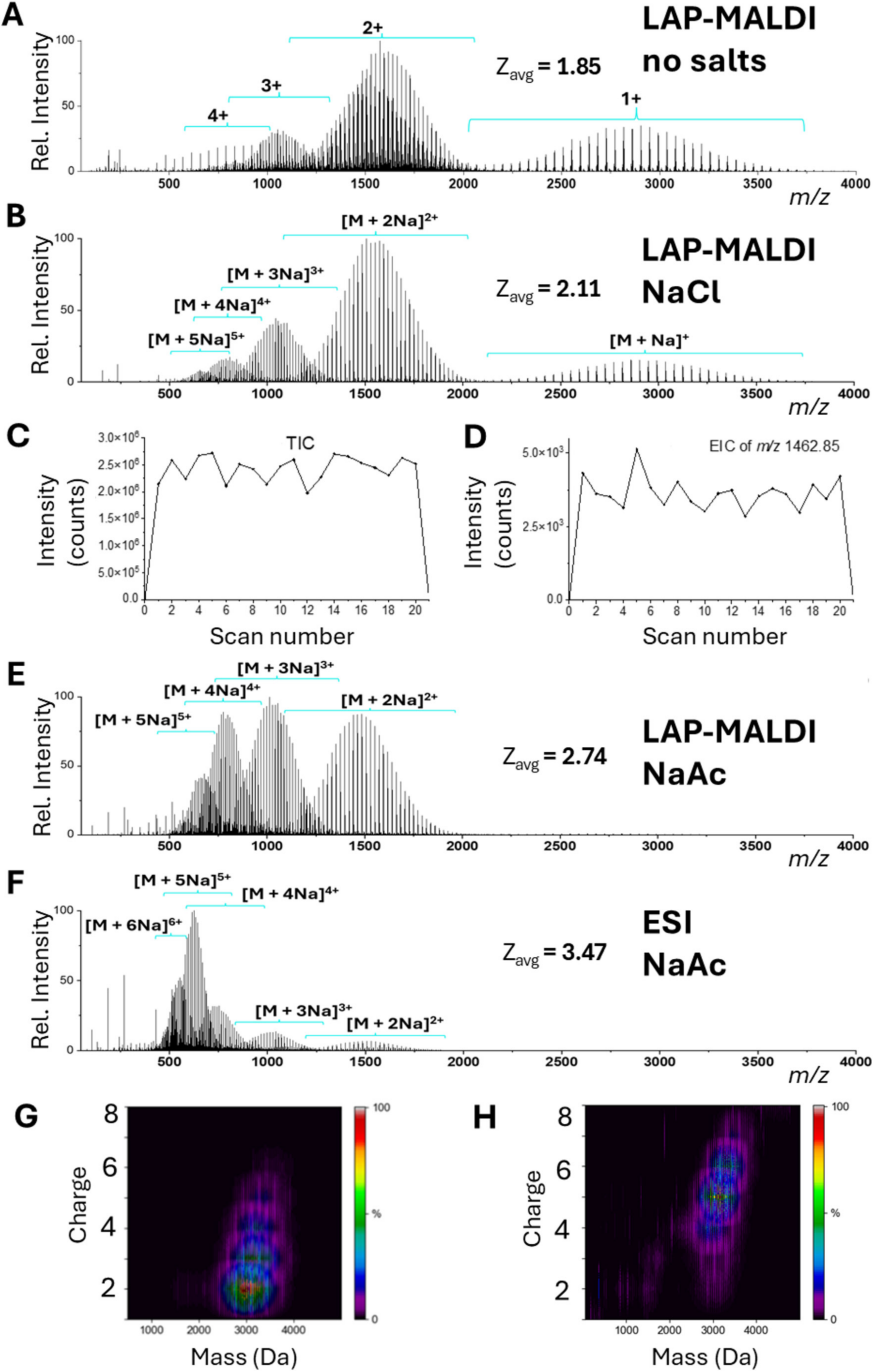
Positive ion mode MS analysis of PEG 3000 (1 mg/mL) using no added salt or sodium salt concentrations of 100 mM. (A) LAP‐MALDI mass spectrum using a CHCA‐based matrix without any salt addition. (B) LAP‐MALDI mass spectrum using a CHCA‐based matrix with sodium chloride. (C) Total ion chromatogram (TIC) for the data acquisition of Panel (B) with a data acquisition rate of 1 Hz. (D) Extracted ion chromatogram for the doubly sodiated PEG ion at *m*/*z* 1462.85 using the same data as Panel (B)/(C) with a data acquisition rate of 1 Hz. (E) LAP‐MALDI mass spectrum using a CHCA‐based matrix with sodium acetate. (F) ESI mass spectrum obtained with sodium acetate. (G) Deconvoluted LAP‐MALDI mass spectrum displayed in Panel (E), showing the relationship between mass and charge. (H) Deconvoluted ESI mass spectrum displayed in Panel (F), showing the relationship between mass and charge.

Figure 1A,B displays LAP‐MALDI mass spectra of PEG 3000 obtained without any salt addition and by adding a salt solution of 300‐mM NaCl, respectively. The latter led to a final NaCl concentration of 100 mM in the LAP‐MALDI sample droplet. As previously shown for protonated peptides and proteins [24], Figure 1 demonstrates that LAP‐MALDI supports the predominant production of multiply charged analyte ions. In agreement with other MS studies of PEG [11, 30], the addition of salts containing metal cations virtually eliminates protonation and replaces it with extensive cationization using the metal cation provided by the chosen salt. Thus, the dominant type of quasimolecular PEG ions after salt addition can be described as [PEG + nMe]^*n*+^ with only a few other PEG ions as [PEG + H + (*n* − 1)Me]^*n*+^ (Me: metal atom of the salts used in this study). As the addition of metal salt simplified the LAP‐MALDI mass spectra of all polymers, subsequent measurements were therefore conducted with the help of an adequate amount of metal salt, typically in the final concentration range of 10–100 mM.

In general, LAP‐MALDI polymer samples made with viscous glycols in the matrix solutions such as propylene glycol as used in this study remain stable for 2–15 min depending on the solvent choices and overall droplet composition. In aqueous LAP‐MALDI sample droplets containing PPG and PEG, evaporation was somewhat slower in comparison to peptide and protein‐containing samples, which can be putatively attributed to their accumulation on the liquid surface and its effect on slowing down solvent evaporation [31]. Evaporation of solvents from the droplet over time can likewise influence both overall ion intensity and the resulting charge state distribution of polymers. However, those changes are typically negligible during analysis times as presented here. Figure 1C,D displays the total ion chromatogram (TIC) and extracted ion chromatogram (EIC) for a 20‐s acquisition from a PEG 3000–containing droplet used for obtaining the spectra in Figure 1B. Both show excellent signal intensities and stability over all scans within 9.5% RSD, which is in line with the levels obtained by ESI sources [32].

To understand more about the charge states acquired, the average charge state *z*_avg_ was calculated using the following formula:

(1) $$z_{\mathrm{avg}}=\frac{\sum_{i=1}^{N}\;z_{i}\omega_{i}}{\sum_{i=1}^{N}\;\omega_{i}}$$

with $\omega_{i}$ as the ion signal intensity summed over all polymer ion peaks with charge $z_{i}$.

While conventional (solid‐state, vacuum) MALDI predominantly produces singly charged polymer ions [11, 33], it is evident that higher charge states are preferentially detected with LAP‐MALDI. For the mass spectrum in Figure 1B, *z*_avg_ was determined to be 2.11.

Different types of salt additives were also tested, with the result that some salts increased the propensity of LAP‐MALDI to produce higher charge states. Figure 1E shows the LAP‐MALDI mass spectrum of PEG 3000 obtained by using a NaAc solution at the same final concentration of 100 mM, leading to a calculated *z*_avg_ of 2.74 with a slight shift of the polymeric distributions to lower *m/z* values. The same salt and final concentration were also used for analyzing PEG 3000 by ESI MS. Figure 1F shows the data obtained with a further substantial increase in the value of *z*_avg_ to 3.47 and a decrease in the *m/z* values of the polymeric distributions for all charge states. These data clearly demonstrate that, similar to (protonated) peptides and proteins, LAP‐MALDI also allows for the detection of multiply charged synthetic polymers, with the advantage of detecting singly charged ions as well as being able to modulate the extent of charging by changing the composition of the added salt.

Synthetic polymers are known to obtain different conformations in a similar manner to the biopolymeric proteins, for example, by forming linear chains or globules [34]. In positive ion mode, polymers such as PEG and PPG are thought to form a “bead‐on‐a‐string” or “helical” [35] conformation where their chains wrap around cations and form complexes through interactions with backbone oxygens. This “stretching” behavior of PEG chains was found to accommodate metal cations for the formation of high charge states in ESI [34, 36]. For polymers with the same repeating unit and end groups but differing length, longer chains resulted in a greater extent in the formation of higher charge states in both LAP‐MALDI and ESI MS (see Figure 1G/H), possibly due to steric reasons. Therefore, lower charge states as in solid MALDI should arguably be preferred for accurate average molecular weight determination of polydisperse polymers. Furthermore, when polydisperse polymers acquire a broad range of charge states, the total ion signal intensity is distributed among multiple overlapping charge envelopes. This reduces the sensitivity of individual charge states and complicates spectral interpretation. The overlap further challenges both manual and automated deconvolution, which increases the uncertainty of the determined molecular mass.

**FIGURE 2:**
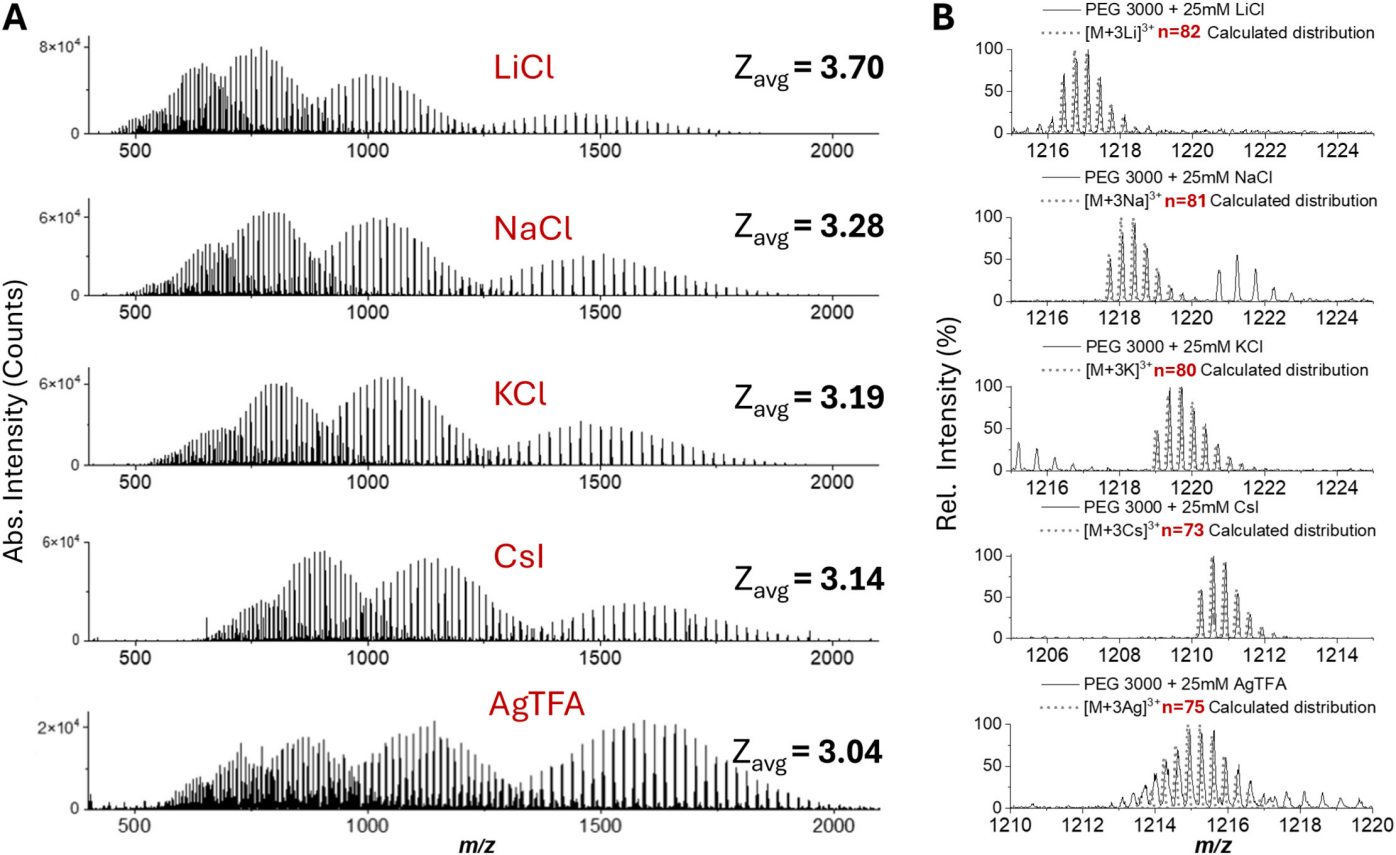
Effect of adding different salts to LAP‐MALDI sample droplets on PEG 3000 (1 mg/mL) ionization and charge state distribution using a DHB‐based matrix. (A) Mass spectra (positive ion mode) using 25‐mM LiCl, NaCl, KCl, CsI, and AgTFA (from top to bottom). (B) Magnified regions of the mass spectra in Panel (A) overlaid with the calculated isotopic distributions of the corresponding triply charged polymer ions.

In this context, LAP‐MALDI MS appears to offer key advantages to ESI MS, afforded by simply changing the composition of the LAP‐MALDI sample droplet. The wide range of salt solutions and sample viscosity that LAP‐MALDI can easily accommodate is often prohibitive in ESI. For instance, by changing the type of salt added to the LAP‐MALDI sample droplet, the value of *z*_avg_ can be substantially changed as shown in Figure 1 and further demonstrated by the PEG 3000 data in Figure 2 using five different salts, all previously used in conventional MALDI MS analyses of synthetic polymers [11, 37]. In Figure 2A, the range for *z*_avg_ is 3.0–3.7 where for the alkali metal ion series the *z*_avg_ value increases with a decrease in ionic radius. In addition, the value of z_avg_ also appears to be dependent on the concentration of the salt as can be seen by comparing the spectrum of Figure 1B with the second spectrum from the top of Figure 2A.

In general, LAP‐MALDI MS analysis of both PEG and PPG exhibited good ion signal intensities using all tested cations, with Na and Cs providing higher spectral simplicity owing to their monoisotopic nature. Theoretical isotopologue distributions for selected PEG polymer ions and the salts used in this investigation are shown in Figure 2B and agree well with the acquired data.

**FIGURE 3:**
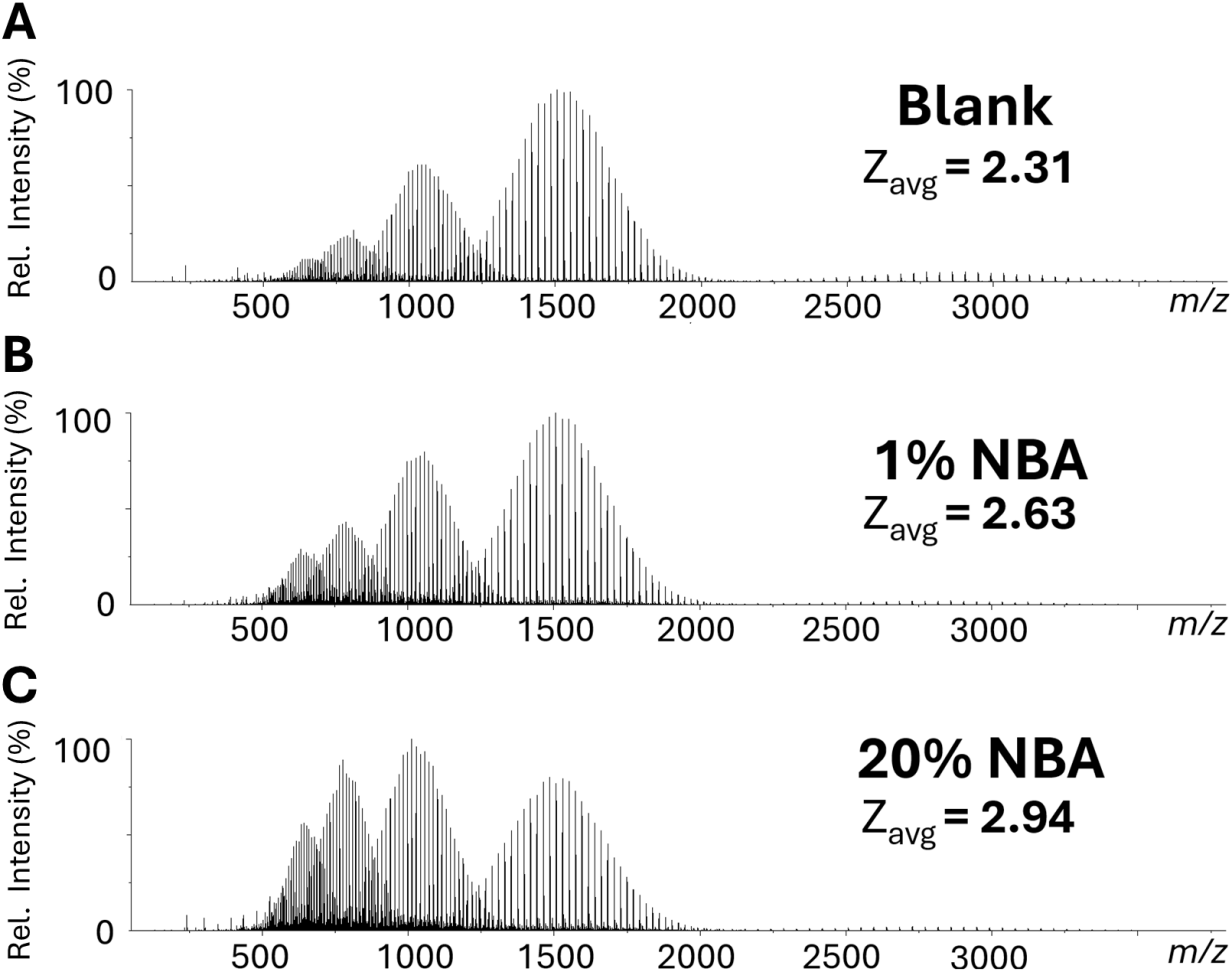
Effect of adding NBA to LAP‐MALDI samples of PEG 3000 (1 mg/mL with 100‐mM NaCl) with respect to ionization and charge state distribution, using a CHCA‐based matrix. (A) Positive ion mode mass spectrum for the addition of 0.5 μL of acetonitrile/water (1:1; v:v). (B) Positive ion mode mass spectrum for the addition of 0.5 μL of 1% NBA in 1:1 acetonitrile/water (1:1; v:v). (C) Positive ion mode mass spectrum for the addition of 0.5 μL of 20% NBA in 1:1 acetonitrile/water (1:1; v:v).

Like the type and concentration of the salt added to the LAP‐MALDI sample, other non‐salt additives can also have significant impact on the detected charge states. Figure 3 shows the effect on *z*_avg_ by changing the concentration of NBA as an additive to the LAP‐MALDI sample droplet. While adding a small volume (0.5 μL) of acetonitrile/water (1:1; v:v) to the sample droplet only slightly increased *z*_avg_, presumably due to the dilution of the NaCl final concentration (cf. Figure 1B vs. Figure 2A), further substantial increases of *z*_avg_ were obtained by increasing the amount of NBA to the acetonitrile/water solution that was added to the sample (see Figure 3A–C).

As before and shown in Figure 3, longer polymer chains resulted to a greater extent in the detection of higher charge states. The maximum detected charge state increased from 5+ to 6+ by the addition of NBA. In previous studies by Iavarone et al. [38], NBA was used as an additive in different solvent formulations to change the degree of charging in ESI MS analysis of PEG. Whether NBA in LAP‐MALDI produces similar effects as observed with these ESI studies needs further investigation and could provide additional evidence for the close relationship of LAP‐MALDI to ESI. Overall, the data from Figures 1, 2, 3 show that LAP‐MALDI with its highly flexible sample preparation allows for a variety of possibilities to change substantially the charge state distributions of PEG.

**FIGURE 4:**
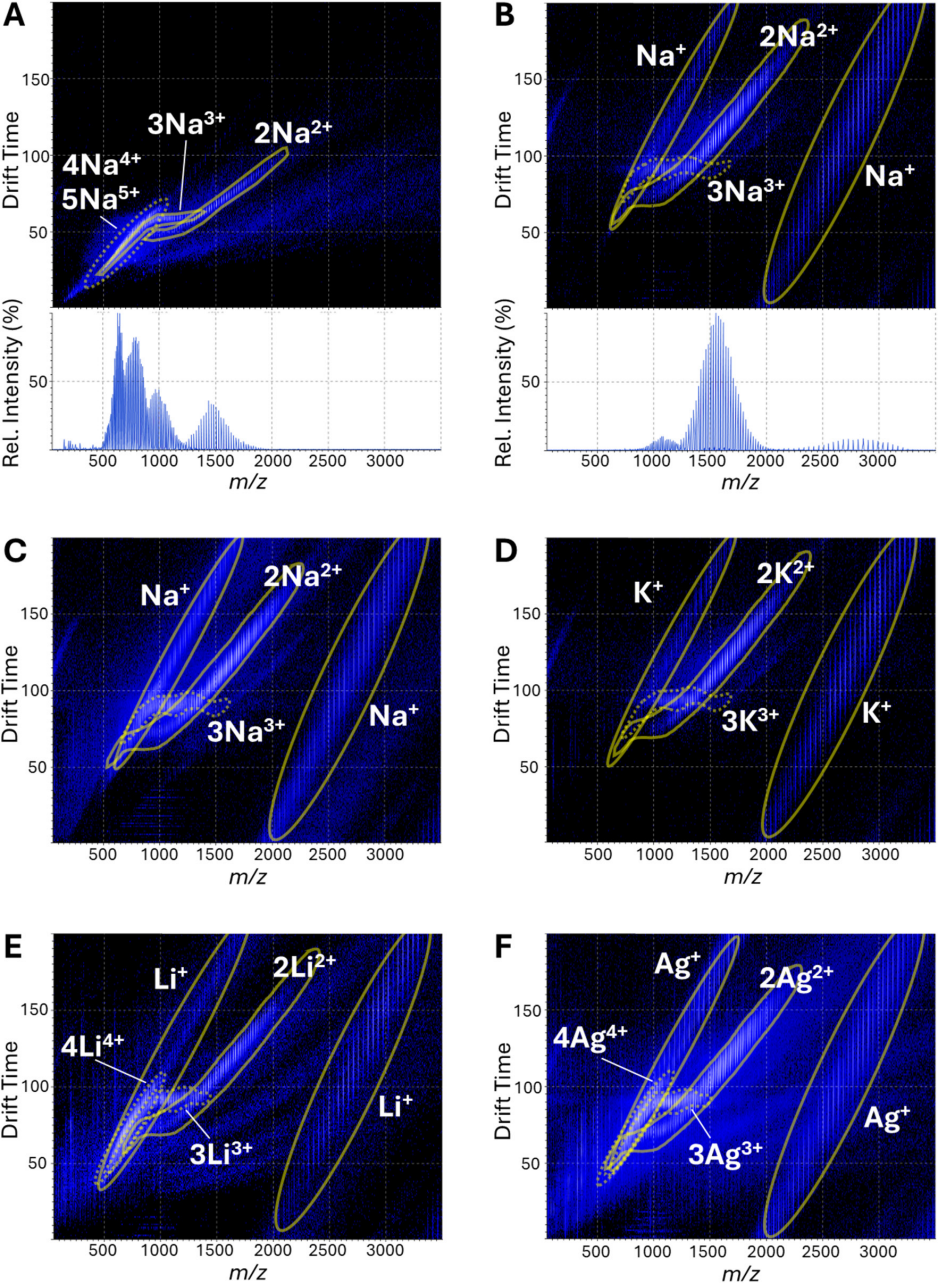
LAP‐MALDI drift time‐versus‐*m*/*z* plots and mass spectra for PEG 3000 (1 mg/mL) using a CHCA‐based matrix. Panel (A) data were acquired using a wave velocity of 414 m/s (IMS cell), 8 m/s (transfer cell), and 221 m/s (trap cell) and wave height of 19.9 V (IMS cell), 3.4 V (transfer cell), and 2.1 V (trap cell). Panels (B)–(F) data were acquired using the IMS parameters as detailed in the methods section. Samples were prepared with (A/B) 100‐mM NaCl, (C) 100‐mM NaCl and 20% NBA, (D) 100‐mM KCl, (E) 100‐mM LiCl, and (F) 10‐mM AgTFA.

As the instrument in this study allows for ion mobility spectrometry (IMS) measurements, a few preliminary IMS analyses were also undertaken. Figure 4 shows for the PEG 3000 sample that polymer distributions can be separated in the drift time‐versus‐*m/z* plots according to their charge state. However, while the drift time of the singly charged PEG species continuously increases with the polymer chain length, distributions of multiply charged PEG ions exhibit *m/z* ranges where an increase in the length of the polymer chain does not result in an increase in drift time. Particularly, the triply charged PEG species show this effect for the *m/z* range of ~800–1600, while this range is smaller and slightly shifted to lower *m/z* values (~750–1100) for the doubly charged species (see Figure 4). For a similar PEG and ESI IMS‐MS analysis, a very similar behavior has been previously reported [39], indicating conformational changes that are charge state‐dependent.

Interestingly, changing the trap, IMS, and transfer cell parameters had a significant impact on the MS detection of the various polymer charge states as can be seen by comparing Figure 4A with Figure 4B. Thus, IMS provides an additional dimension for manipulating the detection of certain charge states. Similar behavior can be expected from ESI‐generated polymer ions and warrant further investigation, which is unfortunately beyond the scope of this study. One main aspect of such investigation should be the potential for charge stripping as revealed by comparing the mass spectra of Figures 1B and 4B, which have been acquired using the same sample preparation and polymer. A previous study [40] using similar instrumentation focused on collision‐induced charge stripping (CICS) at the inlet cone and in the collision cell prior to IMS but not at the IMS stage. However, visualizing all charge states equally using one set of IMS parameters was not achievable.

When IMS parameters were kept constant, no substantial differences in drift times were observed for the individual PEG ion species when the amount of NBA additive (cf. Figure 4B/C) or the type of cation adduct (cf. Figure 4B/D/E/F) was changed.

For the spectra of Figures 1 and 3, the number‐average molecular weight (*M*_*N*_), weight‐average molecular weight (*M*_*W*_), and polydispersity (*PD*) were calculated based on the monoisotopic *m/z* values of the quasimolecular (sodiated) ions, adjusted by subtracting the mass of the relevant number of sodium ions, and are summarized in Table 1. The manufacturer‐provided GPC‐based information for this PEG 3000 is 2591 for *M*_*W*_, 2016 for *M*_*N*_, and 1.29 for *PD*. In all cases, the *PD* value was lower for LAP‐MALDI compared to ESI. The observed differences to the manufacturer‐provided GPC data can be explained by inherent methodological differences between GPC‐ and MS‐based analyses. Since GPC is a comparative analytical method, it has inherent limitations as described in the introduction, in particular for the analysis of smaller polymers. For MS‐based methods, changing ionization efficiencies across the various polymer chain lengths can influence these values. In many cases, GPC‐ and MS‐based methods are ideally used in concert to obtain a more comprehensive understanding of the polymer under investigation.

**TABLE 1:** Polymer characterization values for PEG 3000 as measured by LAP‐MALDI MS and ESI MS for different sample preparations.

	$M_{N}\;\left(\mathrm{Da}\right)$	$M_{W}\;\left(\mathrm{Da}\right)$	PD
LAP‐MALDI (100‐mM NaCl)	3134.99	3170.92	1.01
LAP‐MALDI (100‐mM NaCl and 1% NBA)	3087.93	3138.83	1.01
LAP‐MALDI (100‐mM NaCl and 20% NBA)	3053.61	3134.66	1.03
LAP‐MALDI (100‐mM NaAc)	3145.18	3177.09	1.01
ESI (100‐mM NaAc)	3011.25	3146.87	1.05

Other synthetic polymers that were also analyzed by LAP‐MALDI MS included PPG, PS, and PAA. PPG 2700 and PPG 4000 were less well characterized by the manufacturers than PEG 3000, with neither GPC nor MS data made available.

**FIGURE 5:**
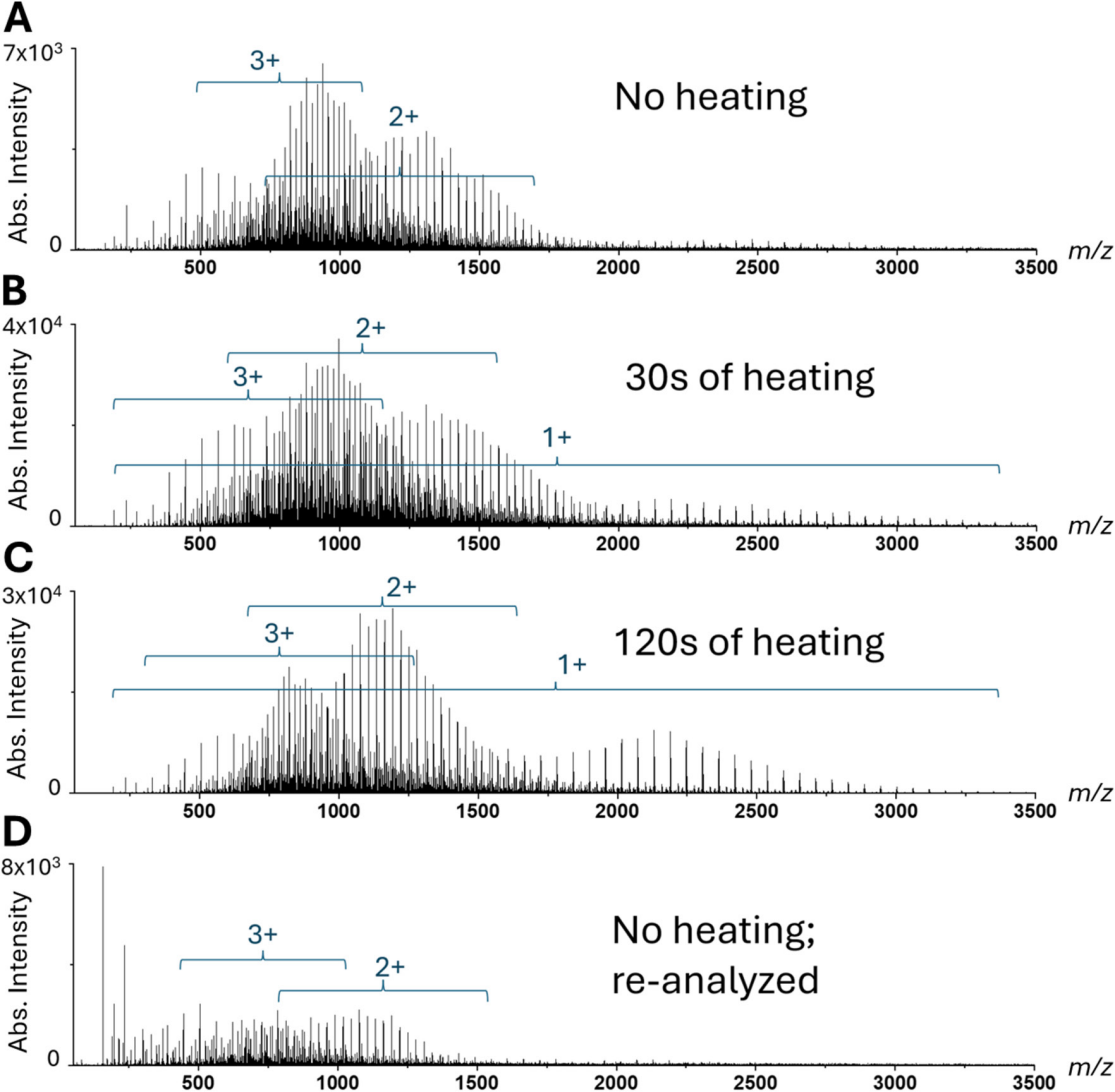
LAP‐MALDI mass spectra generated from a single droplet containing PPG 2700 (1 mg/mL with 100‐mM NaCl) using a CHCA‐based matrix under varied inlet conditions. (A) Capillary heating turned off. (B) After 20–30 s of heating. (C) After 2 min of heating. (D) Capillary heating off and cooled down.

For PPG 2700, LAP‐MALDI MS analysis enabled the detection of abundant singly, doubly, and triply charged PPG ions (see Figure 5). However, only when no heat was applied to the ion transfer tube, mainly multiply charged hydroxyl‐terminated PPG ions (2+ and 3+) were detected (see Figure 5A). As soon as the ion transfer tube was heated, the ion signals of all charge states were increased, with singly and doubly charged ions being then more dominant, indicating the possibility of pyrolysis product formation as previously recorded by MS analysis of PPG samples that were heat‐treated for several hours at 155°C [41]. This temperature value is at the lower end of the temperature range applied to the heated ion transfer tube of the employed ion source, which is generally around 100°C–300°C [26]. All singly charged PEG ion signals increased with increasing temperature on the ion transfer tube, as shown by the mass spectra in Figure 5B/C at two different time points after the resistance wire voltage is applied. Although a heated ion transfer tube is typically essential for the generation of multiply protonated peptide and protein ion signals [24], it appears that for metal cationization of synthetic polymers this is not necessarily the case—potentially owing to a different ionization process behind metal cationization of some synthetic polymers and/or differences between the availability of metal cations and protons at various places and times throughout the desorption process or path to the mass analyzer.

Whether the heated ion transfer tube supports pyrolysis is currently unclear. However, as the increased singly charged PPG ion signals display a bimodal distribution, two different effects could be present when the ion transfer tube is heated. First, a general increase of metal cationization, in particular of singly charged (intact) polymer ions, potentially due to gas‐phase ionization of polymer molecules by thermally desorbed metal cations, which can be omnipresent on all interior instrument walls along the path to the mass analyzer. Second, an increase of shorter polymer ions due to pyrolysis. Arguably, both effects together could result in a bimodal distribution as observed. Interestingly, no substantial bimodal distributions of PPG ions were recorded for all multiply charged PPG ions (see Figure 5B/C). Furthermore, using the same sample that was previously analyzed by LAP‐MALDI MS with a heated ion transfer tube and then reanalyzed without heating the ion transfer tube virtually reverts back to the ion signal and charge state distribution observed initially without heating the ion transfer tube (cf. Figure 5A,D), indicating insignificant pyrolysis within the LAP‐MALDI sample droplet, and thus excluding substantial pyrolysis as a result of the employed laser irradiation.

As a cautionary measure, it is therefore advisable for thermally labile polymers to avoid a heated ion transfer tube that could potentially induce pyrolysis, which could have a direct impact on accurate average molecular weight determination.

For PPG 4000, LAP‐MALDI and ESI MS analysis revealed an abundance of low‐molecular mass, singly charged polymers at *m/z* values of 255, 313, 371, 429, 487, and so on, indicating an oxypropylene repeating unit with a mass of ~58 Da. These singly charged ion signals were largely independent of heating the ion transfer tube, with a slightly higher average molecular mass when the ion transfer tube was heated, also compared to the ESI MS data. In this case, it is assumed that no pyrolysis takes place, and the heated ion transfer tube rather promotes ionization as indicated by the increased ion signals of the longer chain polymers. Although in some cases singly charged polymer peaks were detected beyond *m/z* 2000, all average molecular weights were below 1000. An abundance of these singly charged polymer peaks was previously reported in an ESI MS study of commercial PPG 3000 and putatively assigned to sodiated cyclic or vinyl‐terminated linear oligomers [42]. Barton et al. [41] assigned similar (protonated) ion signals to the formation of [HO‐[CH_2_‐CH(CH_3_)‐O]_n_‐CH_2_‐CH(CH_3_)]^+^ products, which in this present study would be sodiated and most likely already existent in the prepared sample.

**FIGURE 6:**
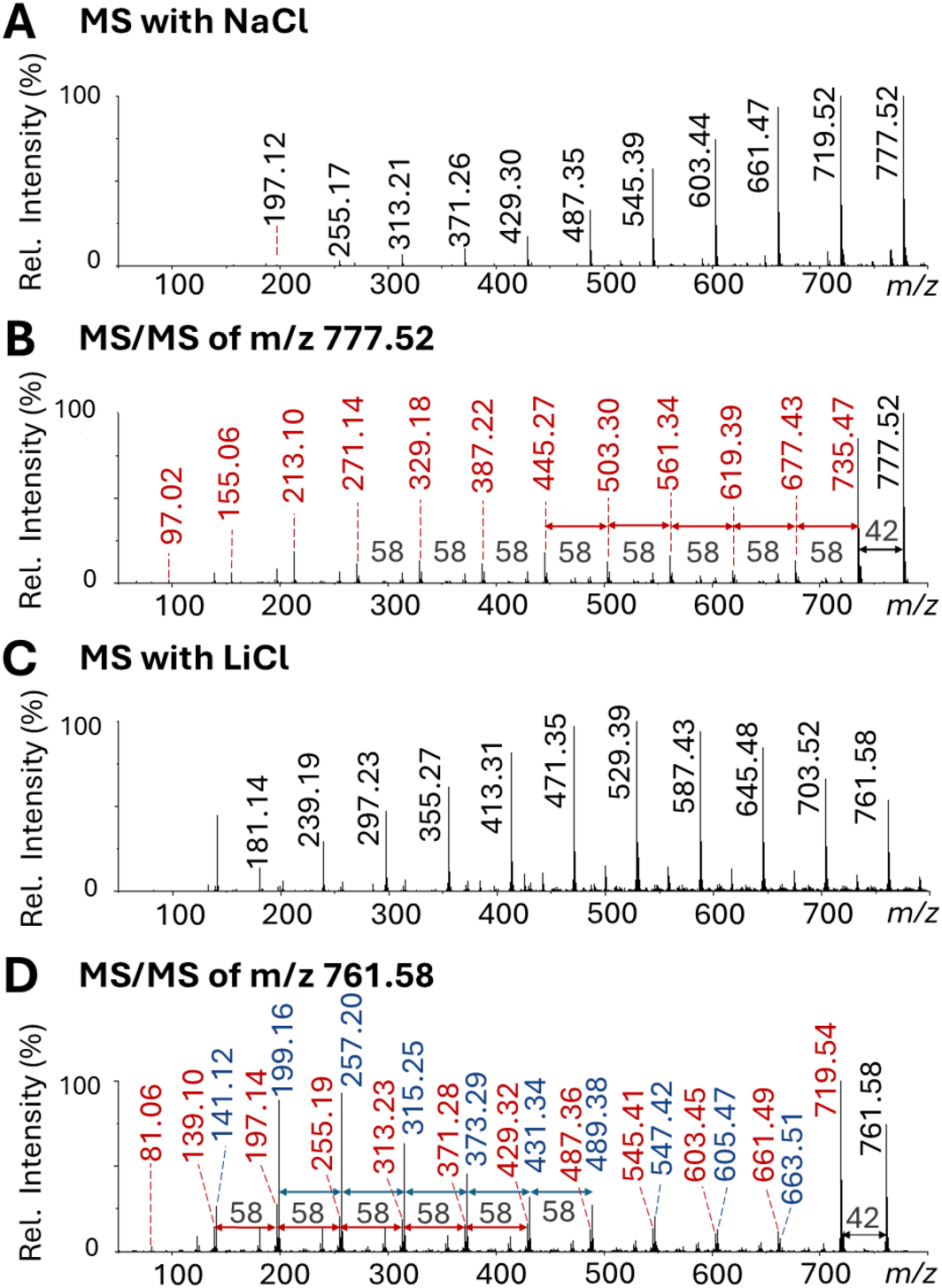
LAP‐MALDI mass spectra of PPG 4000 (1 mg/mL) showing (A) sodiated MS profile below *m*/*z* 800; (B) MS/MS spectrum of the singly charged, sodiated precursor ion at *m*/*z* 777; (C) lithiated MS profile below *m*/*z* 800; and (D) MS/MS spectrum of the singly charged, sodiated precursor ion at *m*/*z* 761.

Further structural characterization of these PPG ions was obtained by MS/MS analysis (see Figure 6), which suggests the neutral loss of CH_2_=CH(CH_3_) (~42 Da) with subsequent losses of the repeating unit (~*n*·58 Da). Given the MS/MS data for LiCl (Figure 6D), these low molecular weight PPG ions can be explained by HO–[CH_2_–CH(CH_3_)–O]_n_–CH=CH(CH_3_) PPG, which leads to fragment ions of the form [HO–[CH_2_–CH(CH_3_)–O]_m‐1_–CH_2_CO(CH_3_) + Li]^+^ (*m* ≤ *n*) as observed among the larger fragment ions (e.g., *m/z* 719, 661, 603, 545, and 487) and [HO–[CH_2_–CH(CH_3_)–O]_n‐1_–H + Li]^+^ (*m* ≤ *n*) as predominantly observed among the smaller fragments ions (e.g., *m/z* 141, 199, 257, 315, and 373). These assignments are in agreement with the ESI MS/MS studies by Jackson et al. that were undertaken using the same PPG concentration and lithium acetate [43].

**FIGURE 7:**
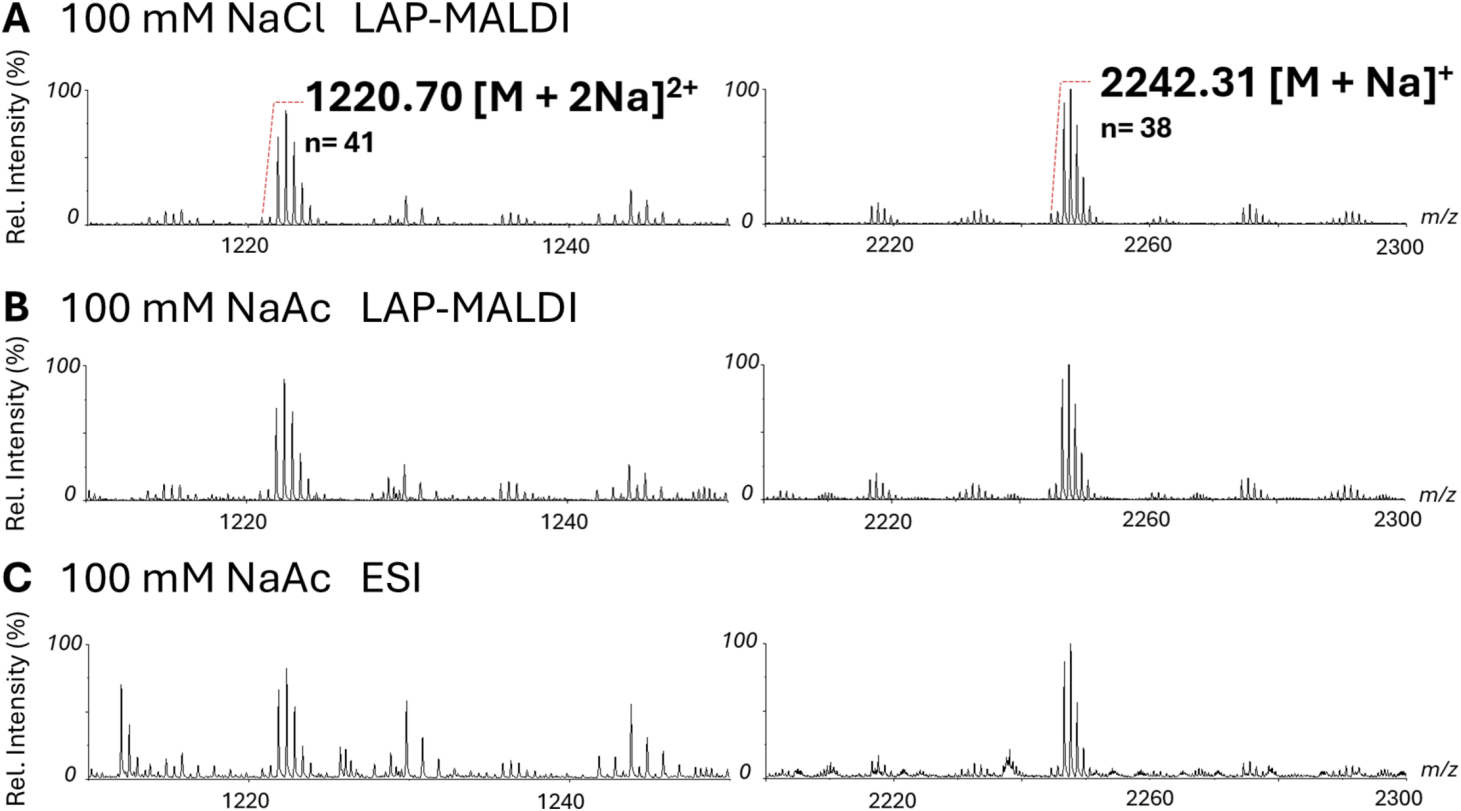
Zoomed‐in mass spectra of 1‐mg/mL PPG 2700 showing differences in background ion signals in doubly and singly charged regions for (A) LAP‐MALDI with 100‐mM NaCl, (B) LAP‐MALDI with 100‐mM NaAc, and (C) ESI with 100‐mM NaAc.

Less abundant multiply charged ion signals of dihydroxyl‐terminated PPG for higher molecular polymer masses were also obtained for PPG 4000. Interestingly, for both PPG 2700 and PPG 4000, ESI MS provided higher absolute polymer ion signals, particularly for the lower mass (shorter chain) polymers, but also greater background ion signal, mainly due to multiply charged ions, which can easily overlap. An extensive overlap of various multiply charged ions can lead to distortions in the determination of accurate masses as well as the average molecular weight distribution and, thus, is generally undesirable. Again, LAP‐MALDI MS and its flexible sample preparation can reduce extensive multiply charged ion production and lead to a simpler and cleaner background as shown in Figure 7.

**FIGURE 8:**
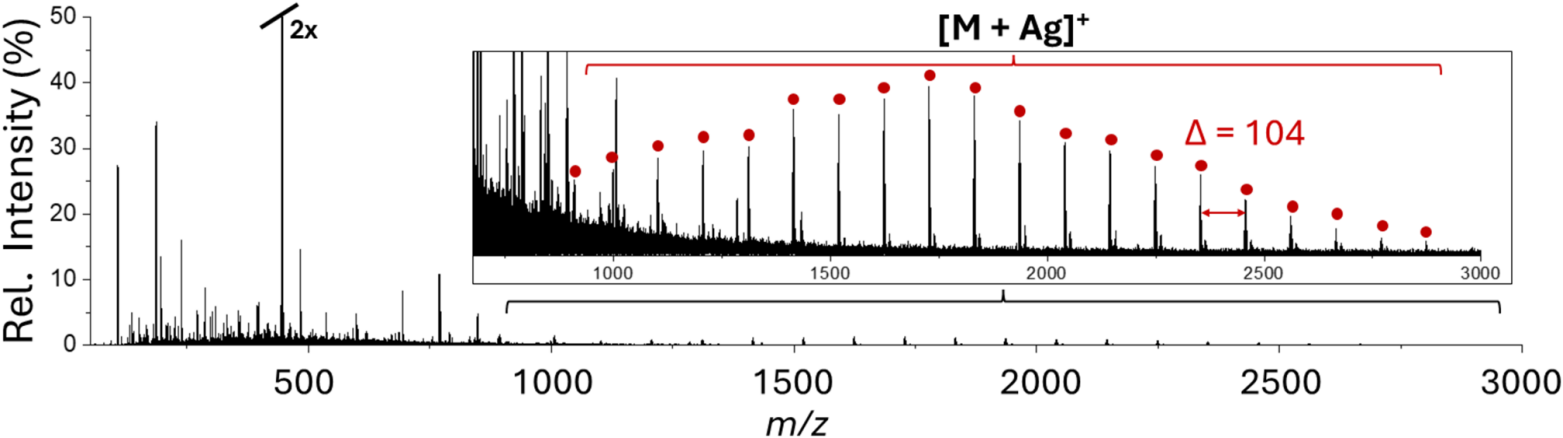
LAP‐MALDI mass spectrum of PS 3000 acquired from a 1‐μL droplet prepared by mixing 0.5 μL of 2‐mg/mL PS 3000 in THF containing 10‐mM AgTFA with 0.5 μL of 10‐mg/mL dithranol in THF:DMSO (1:1; v:v) as the liquid matrix.

While alkali metals are known to interact electrostatically with oxygen‐containing species such as polyesters and polyethers, transition metal cations such as Ag^+^ or Cu^+^ are known to bind strongly to *π* bond‐containing polymers such as polystyrene or polyolefins [44, 45]. Therefore, AgTFA was employed for LAP‐MALDI MS analysis of PS 3000 as shown in Figure 8. Furthermore, dithranol, a solid MALDI matrix previously employed for polymer analysis [46], was used as the chromophore as it was superior in the detection of polystyrene compared to CHCA and DHB. Since PS is non‐polar, aprotic THF was employed for the analyte solution and matrix formulation. The disadvantage of using THF in LAP‐MALDI is its higher volatility compared to more aqueous/polar solvents, resulting in LAP‐MALDI sample droplet evaporation within seconds. These formulations therefore benefitted from the addition of DMSO, up to 25% of the LAP‐MALDI sample droplet content, where the sample droplet was too volatile and insufficiently stable. The addition of DMSO was found to extend the droplet lifetimes to several hours under ambient conditions. As shown in Figure 8, LAP‐MALDI mass spectra of PS 3000 predominantly displayed [M + Ag]^+^ ions with a spacing between peaks of *m/z* 104. Low ion signals of [M + 2Ag]^2+^ ions were also observed, but these were too weak to provide useful information.

**FIGURE 9:**
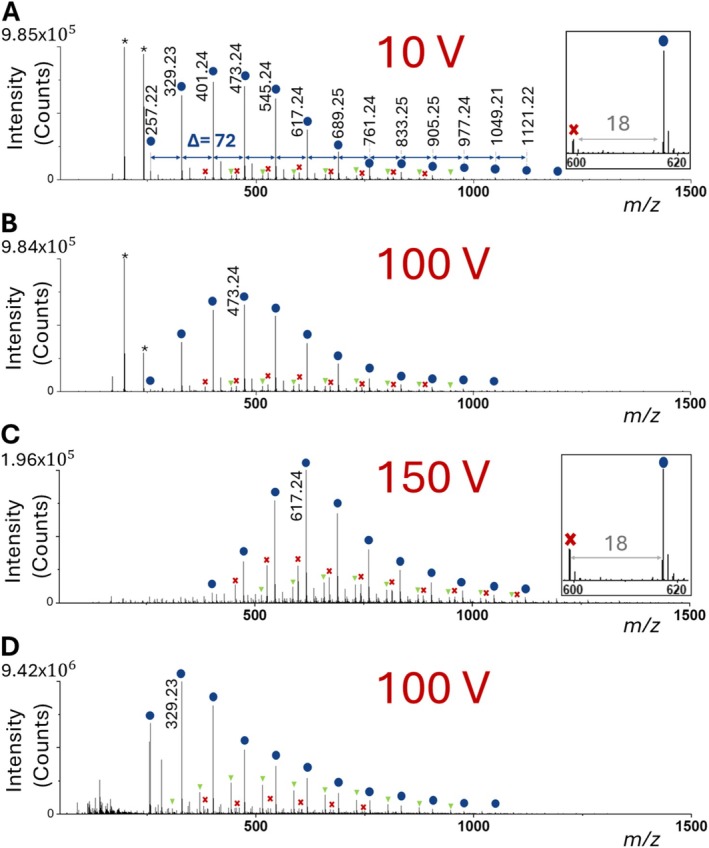
Negative ion mode mass spectra of PAA. The main PAA ion signals can be attributed to [PAA ‐ H]^−^ ions with cyclized C_6_O_2_H_9_ and hydrogen end groups (*M*_*W*_  O≈ (*n* − 1)·72 + 114 Da; circles). For LAP‐MALDI, 0.5 μL of PAA solution (2 mg/mL) was mixed on target with 0.5 μL of HABA matrix solution without any addition of a salt solution. The spectra were recorded at different cone voltages: (A) 10, (B) 100, and (C) 150 V. (D) ESI mass spectrum acquired with a cone voltage of 100 V. The insets in (A) and (C) show the change in water loss (−18 Da) with increased cone voltage. Minor PAA series are labeled with crosses and triangles, and matrix peaks are labeled with an asterisk.

For the analysis of PAA, its functional groups are easily deprotonated, thus promoting their detection as [PAA ‐ H]^−^ ions in negative ion mode, as seen in Figure 9. The ion signal distribution of the detected PAA peaks was found to be dependent on the set cone voltage (Figure 9A–C), with lower values in the range of 0‐50 V resulting in relatively higher ion signal intensities of the lower molecular weight chains. This was found in contrast to the analysis of PEG and PPG, where the cone voltage parameter did not affect the overall peak distribution. A cone voltage of 100 V provided the highest individual PAA ion signals, with values above 100 V resulting in an overall drop of the PAA (and other) ion signals, possibly due to in‐source fragmentation and/or unfavorable ion extraction and transfer.

PAA was reported to thermally decompose starting slightly above 100°C [47], giving rise to mainly dehydration, and at higher temperature starting at 200°C, also decarboxylation products [48]. However, the PAA analyzed in this study using a heated ion transfer tube with temperatures in the range of 100°C–300°C resulted in ion signals similar to those obtained for PAA by a previous study without heat treatment using conventional (solid, vacuum) MALDI MS [48], showing no elevated loss of water or decarboxylation, which were evident in this previous study after heat treatment in the range of 175°C–225°C. Therefore, the lack of previously described degradation products of heat‐treated PAA suggests that thermal degradation is a minor concern for PAA analysis by LAP‐MALDI MS. Since some additional loss of water is observable at the higher cone voltage of 150 V (Figure 9C), degradation due to in‐source collisions cannot be excluded. Other smaller polymeric ion signals were also seen in the LAP‐MALDI mass spectra of PAA and can be assigned to PAA polymers with different end groups similar to the ones described by Lattimer et al. [48] in their PAA study.

The ESI mass spectrum of PAA (Figure 9D) essentially displays the same type of PAA ion signals as the LAP‐MALDI mass spectra. In all cases, PAA ion signals are substantially reduced above *m*/*z* 1000, and their distribution is visibly unsymmetrical with an elongated slope of reduction toward the higher *m*/*z* values and a much steeper slope toward the lower *m/z* values. Similar spectra were previously reported for PAA using ESI and MALDI MS [48]. However, the ESI mass spectrum shown in Figure 9D shows higher (relative) PAA ion signals in the lower *m/z* range than the LAP‐MALDI mass spectra. In addition, esterification as described by Lattimer et al. [48] appears to be more pronounced in ESI than LAP‐MALDI (triangles in Figure 9). Thus, the extent of the recorded different PAA species and their ion signal distributions is slightly different between ESI and LAP‐MALDI MS, but the main ion signal series and its distribution is very similar and in agreement with the literature.

The exact process of multiply charged ion generation with LAP‐MALDI still remains unclear. However, by using the above instrumental setup with a heated ion inlet, together with a liquid sample formulation, the underlying ionization processes appear to be distinctly different compared to other atmospheric pressure methods. Ion formation under atmospheric or near‐atmospheric pressure conditions has also been described for related techniques such as laser‐based solid AP‐MALDI, MALDESI [49], LAESI [50], LSI [51], or non‐laser‐based techniques such as SAI [52], MAI/MAIV, and plasma‐driven DART [53]. Some of these approaches also lead to multiply charged ion formation and similarly invoke combinations of laser‐ and/or matrix‐assisted desorption, droplet‐mediated processes, and gas‐phase ion‐molecule interactions to explain the generation of highly charged ion species.

In the case of multiply charged ions obtained with LAP‐MALDI, they are hypothesized to be driven by a “hybrid” of conventional MALDI‐ and ESI‐like processes. During charge build up in the LAP‐MALDI sample droplets through the applied high extraction potential (3–4 kV), laser desorption/ablation is supported by laser energy absorption of an adequately high concentration of matrix chromophores in the irradiated sample volume. The post‐desorption/ablation events leading to ESI‐like quasimolecular ions are more speculative and could occur during UV‐laser irradiation, via chromophore‐analyte charge transfer, movement of the plume through the extraction field region, and later by ion/molecule charge transfer and collisions in and/or with the heated inlet. The latter is of particular relevance to the ionization of synthetic polymers by metal cationization, as metal cations from previously desorbed sample volumes and deposited on the interior walls of the ion inlet components can easily be thermally desorbed and be available for ionization of neutral polymers in these regions. Such additional ionization processes are heat‐dependent and would favor singly charged ion production, which could explain the differences seen in the relative abundance of the singly charged ion species of PPG 2700 that were obtained with and without heat applied to the ion transfer tube.

## 4 Conclusions

This study demonstrates that LAP‐MALDI is a new, versatile tool for the characterization of synthetic polymers. Although there are common features between conventional MALDI and LAP‐MALDI (laser‐based, fast MS analysis with similar energy deposition), and between ESI and LAP‐MALDI (production of mainly multiply charged polymer ions), there are also distinct differences and therefore opportunities using LAP‐MALDI for the MS analysis of synthetic polymers. First, LAP‐MALDI MS analysis of synthetic polymers results in lower average charge states than ESI MS and therefore less convoluted mass spectra, making the mass measurement of individual ion peaks and average molecular weight distributions potentially more accurate. Second, the flexibility and versatility of LAP‐MALDI sample preparation allow for sample compositions with additives such as metal salts, solvent additives such as NBA and combinations of these that can support the detection and characterization of synthetic polymers under conditions that might be incompatible with ESI MS analysis. Third, instrumental parameters such as the inlet temperature and cone potential can further tune the charge state and/or average molecular weight distribution and potentially support fragmentation and thermal degradation studies at speed and in a controlled way. Fourth, lower *m/z* values as a result of the higher charge states compared to conventional MALDI MS analysis and the use of high‐performance hybrid mass spectrometers, like Orbitraps and Q‐TOF instruments as used in this study, enable higher separation and mass accuracy as well as superior MS/MS capabilities than shown with conventional MALDI MS analysis of singly charged synthetic polymers on axial TOF mass spectrometers.

In summary, this first study of investigating LAP‐MALDI for the analysis of synthetic polymers revealed a set of new and distinct MS‐based analytical possibilities, which can complement and add to the capabilities of conventional MALDI and ESI MS analysis of synthetic polymers. Further optimization of instrumental parameters, droplet compositions, and additive strategies could expand its use across a wider range of applications and systems in synthetic polymer research and analysis.

## Author Contributions

**Agata E. Kowalczyk:** formal analysis, investigation, methodology, writing – original draft, writing – review and editing. **Jeffery Brown:** resources, methodology, writing – review and editing. **Michael Morris:** resources, writing – review and editing. **Rainer Cramer:** conceptualization, investigation, funding acquisition, writing – original draft, writing – review and editing, formal analysis, supervision, validation, methodology.

## Funding

This work was supported by the Waters Corporation and the University of Reading.

## Data Availability

Data supporting the results reported in this paper are openly available from the University of Reading Research Data Archive at https://doi.org/10.17864/1947.001515.
